# Piezoresistive AFM cantilevers surpassing standard optical beam deflection in low noise topography imaging

**DOI:** 10.1038/srep16393

**Published:** 2015-11-17

**Authors:** Maja Dukic, Jonathan D. Adams, Georg E. Fantner

**Affiliations:** 1 Laboratory for Bio- and Nano-Instrumentation, École Polytechnique Fédérale de Lausanne, Batiment BM 3109 Station 17, 1015 Lausanne, Switzerland

## Abstract

Optical beam deflection (OBD) is the most prevalent method for measuring cantilever deflections in atomic force microscopy (AFM), mainly due to its excellent noise performance. In contrast, piezoresistive strain-sensing techniques provide benefits over OBD in readout size and the ability to image in light-sensitive or opaque environments, but traditionally have worse noise performance. Miniaturisation of cantilevers, however, brings much greater benefit to the noise performance of piezoresistive sensing than to OBD. In this paper, we show both theoretically and experimentally that by using small-sized piezoresistive cantilevers, the AFM imaging noise equal or lower than the OBD readout noise is feasible, at standard scanning speeds and power dissipation. We demonstrate that with both readouts we achieve a system noise of ≈0.3 Å at 20 kHz measurement bandwidth. Finally, we show that small-sized piezoresistive cantilevers are well suited for piezoresistive nanoscale imaging of biological and solid state samples in air.

Of the methods proposed to detect cantilever deflection in atomic force microscopy (AFM)^1^, the optical beam deflection method (OBD)^2^,^3^ remains predominant, due to its low noise, its reliability and its ability to use a variety of cantilever sensors. OBD readout uses a focused laser beam to measure cantilever angular changes caused by deflection of the cantilever tip (see Fig. 1a). The laser beam reflects from the cantilever surface towards a position sensitive detector, where a shift in the laser spot position is measured. Further signal processing is usually achieved by using a transimpedance amplifier and voltage arithmetic electronics^4^,^5^.

The OBD method also has certain limitations, including a cumbersome measurement setup requiring frequent laser alignment and a cantilever with a reflective surface, and imaging artefacts due to stray light reflected by the sample surface. The latter phenomenon is a problem particularly in high quality metrology applications for the semiconductor industry^6^. Furthermore, due to the optical diffraction limit, only cantilevers with widths down to a few micrometres are usable for imaging. It is well-known that a reduction in cantilever size increases both sensitivity and detection speed^7^,^8^, and this diffraction limit presents a major barrier for the use of OBD readout with increasingly miniaturized cantilevers. Beyond the OBD technique, many other deflection sensing techniques were proposed in the past: capacitive^9^,^10^,^11^,^12^,^13^, doped silicon and polysilicon piezoresistive^14^,^15^,^16^,^17^,^18^,^19^,^20^,^21^,^22^, piezoelectric^23^,^24^,^25^,^26^, magnetic^27^,^28^ and thin metal film^7^,^29^,^30^ deflection sensing. Techniques with strain-sensing elements incorporated in the cantilever are of a particular interest, offering several advantages over external readout techniques^31^. These include a compact measurement setup that occupies little space and allows for integration in large cantilever arrays, imaging in environments with low or varying optical transparency, imaging of samples with geometrical constraints, imaging of light-sensitive samples, and potential to detect submicron-sized cantilevers^7^.

Initial piezoresistive self-sensing cantilevers primarily used doped silicon resistors^14^,^15^,^17^,^19^,^20^,^21^,^22^, followed by cantilevers with polysilicon^32^,^33^,^34^,^35^ and thin metal film^7^,^29^,^30^ strain-sensing resistors. Piezoresistors measure strain through a change in resistivity (effect dominant in semiconductors) and a change in geometry (effect dominant in metals). Piezoresistive cantilevers are made using standard silicon manufacturing processes and can use a simple measurement setup. The sensors are usually configured in a Wheatstone bridge with differential amplification, where the measured voltage is directly proportional to the cantilever deflection (Fig. 1b). Concurrently to piezoresistive cantilevers, piezoelectric self-sensing cantilevers using various materials (PZT, ZnO, AlN) were developed^23^,^24^,^25^,^26^. Numerous other self-sensing techniques were also demonstrated in the past^36^,^37^,^38^,^39^,^40^, although, none became preferable over optical sensing in routine AFM imaging, due to the comparatively lower signal-to-noise ratio (SNR), detection speed or complexity of integration.

Most self-sensing techniques have been applied on large cantilevers equally well suited for optical readout (in 100s of micrometres) or with cantilever dimensions optimized for force sensing and softer imaging. Although strain-sensing techniques are less suitable for force sensing, they are very well suited for achieving high topography resolution. These large piezoresistive self-sensing cantilevers even achieved low noise imaging^14^,^16^, although with low bandwidth and higher cantilever heating. With the continuing reduction of cantilever sizes to the range of tens of micrometres, the performance of strain-sensing methods in deflection sensing drastically increases. In this work, we show both theoretically and experimentally that smaller size piezoresistive cantilevers permit AFM imaging with noise equal or lower than with OBD readout. We performed a comparison of the imaging noise achievable with the OBD and the piezoresistive readout, in an amplitude modulation AFM (AM-AFM) mode in air on a commercial AFM system. At 20 kHz measurement bandwidth, with both readout techniques, we achieve a deflection noise of ≈0.3 Å, which is the noise level specified for this specific commercial AFM instrument. Finally, we demonstrate that the piezoresistive cantilevers are suitable for nanometre and angstrom scale imaging of solid state samples or even biological samples in air, at standard AFM imaging scan rates.

## Optical beam deflection readout measures angle, piezoresistive readout measures strain

A fundamental difference between OBD and strain-sensing readout is the way they measure cantilever deflection. An OBD readout measures cantilever deflection through angular changes (see Fig. 2a). If a cantilever free end deflection Δ*z* produces an angular change *θ* at the laser beam position (*x* = *l* − *l*_*b*_/2, where *l* is the cantilever length and *l*_*b*_ is the diameter of the laser beam, along the cantilever length), then the signal measured by the optical readout will be proportional to 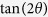 where


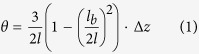


(see Supplementary Section I). For small bending angles, 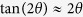. Also, if 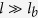 the term in the brackets can be neglected. However, this term should be taken into account if cantilever dimensions become on par with laser spot dimensions (10s of microns).

A strain-sensing readout measures cantilever deflection through the strain induced in the sensor. An average longitudinal strain in a sensor positioned at the cantilever fixed end (see Fig. 2b), induced by the cantilever free end deflection Δ*z* can be approximated as^41^,^42^,^43^


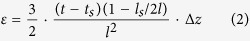


where *l* and *t* are the cantilever length and thickness; and *l*_*s*_ and *t*_*s*_ are the piezoresistor length and thickness.

From equations (1) and (2), one can conclude that changing the cantilever length adjusts the sensitivity of both optical and piezoresistive readout. Specifically, a length decrease will increase the measured signal for a given displacement in both readouts. Figure 3 shows the relative change in tan(2*θ*) and *ε* for varied cantilever length and thickness. A decrease in cantilever length has a much higher impact on piezoresistive readout performance than on OBD performance.

Additionally, from equation (2) we notice that increasing the cantilever thickness improves the deflection sensitivity of piezoresistive readout but does not affect the sensitivity of OBD readout. Based on the results in Fig. 3, we conclude that decreasing the cantilever length and increasing the cantilever thickness significantly improve the deflection sensitivity of piezoresistive readout, but only marginally improve the deflection sensitivity of OBD readout. However, decreasing the length and increasing the thickness also strongly increases the cantilever spring constant. For AFM applications that require soft cantilevers, increasing the deflection sensitivity at the cost of a higher spring constant is not suitable. For AFM imaging applications such as AM-AFM mode in air however, high spring constants of 10s of N/m are used to overcome surface adhesion due to the absorbed water layer. In this application, miniaturized piezoresistive cantilevers can perform very well.

## Noise sources in the cantilever deflection measurements

We classify noise sources in both OBD and strain-sensing readouts into three main groups: noise coming from the actual motion of the cantilever, noise coming from the measurement principle, and noise coming from the readout electronics. In each readout, these noise sources in combination determine the minimum detectable deflection (MDD), which is the deflection that causes the output voltage of the readout to be equal to the root mean square (RMS) voltage noise^44^.

**Figure 1:**
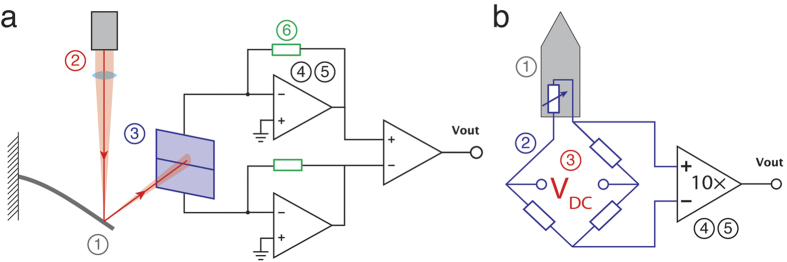
A schematic representation of the measurement setup and the major noise sources present in (**a**) OBD readout and (**b**) piezoresistive readout. (**a**) In OBD readout the major noise sources are: 1. Cantilever thermomechanical noise 2. Laser noise 3. Photodiode shot noise 4–5. Voltage and current noise of an amplifier 6. Noise of a feedback resistor. (**b**) In piezoresistive readout the major noise sources are: 1. Cantilever thermomechanical noise 2. Wheatstone bridge resistor noise 3. Bridge voltage reference noise 4–5. Voltage and current noise of a differential amplifier.

**Figure 2:**
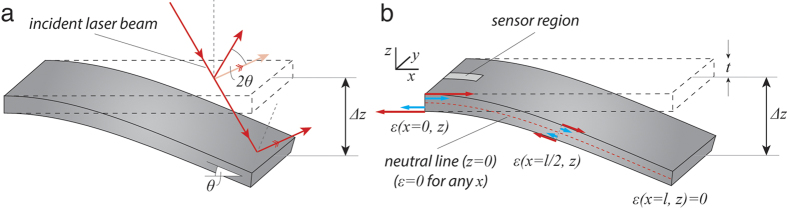
A schematic illustration of (**a**) OBD and (**b**) piezoresistive sensing principles. (**a**) OBD readout measures changes in the bending angle. Upon cantilever deflection, the laser spot will shift towards the cantilever free end. Also, the distance travelled by the laser will change due to Δ*z*. However, in most cases these effects are negligible. The most important effect is the change of the angle of the reflected laser beam, equal to 2*θ*, where *θ* is the cantilever bending angle at the laser spot position. The laser spot should be positioned close to the cantilever free end, where the change in the angle is the highest. (**b**) Piezoresistive readout measures changes in the induced strain. The strain is always zero on the cantilever neutral line (dashed red line), along the whole cantilever length. The strain varies linearly along the cantilever thickness, with maximum compressive strain at the bottom, zero strain at the neutral line and maximum tensile strain at the top (coloured arrows). Along the cantilever length, strain also varies linearly from a maximum at the cantilever fixed end to zero at the cantilever free end (coloured arrows). Therefore, the piezoresistor should be positioned at the regions of maximum strain – the top or bottom surface of the cantilever fixed end.

**Figure 3:**
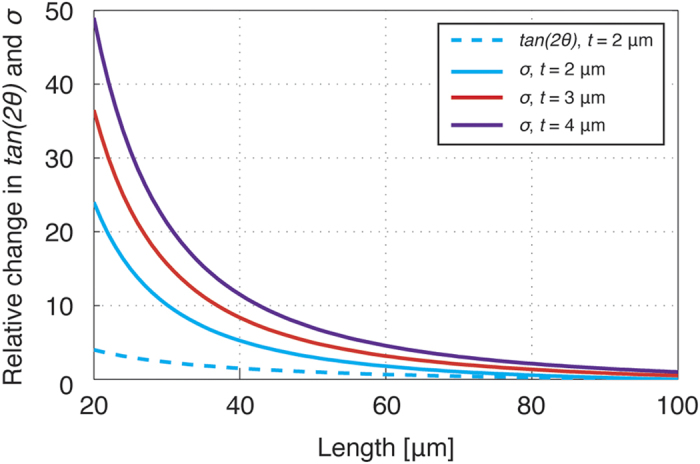
Effect of cantilever dimensions on the maximal bending angle (at *x* = *l*) and maximal strain (at *x* = 0) induced in the cantilever. The figure shows the relative change in cantilever bending angle: 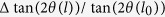, where *l*_0_ = 100 μm and the relative change in generated strain: 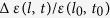, where *l*_0_ = 100 μm, *l*_0_ = 2 μm. A decrease in cantilever length and increase in cantilever thickness will increase strain, and hence performance, of piezoresistive readouts by over an order of magnitude. At the same time, in OBD readouts, performance increase is only a few fold.

Brownian motion causes spontaneous oscillations in microcantilevers, such that each mode of the cantilever oscillation has the same average thermal energy 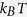
45. These thermal fluctuations are referred to as the thermomechanical noise. Figure 1a,b indicates this noise with number 1. Butt and Jaschke^46^ derived the mean square deflection at the free end of the cantilever for each oscillating mode:


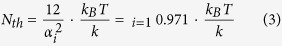


where *k* is the cantilever spring constant, 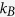 is the Boltzmann constant, *T* is the temperature and 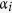 is a constant that is different for each oscillating mode. This noise scales slightly differently in piezoresistive and OBD readouts due to the measurement of displacement as an angle (OBD) or displacement as strain (piezoresistive); for details, see Supplementary Sections IV and VI.

Noise coming from the measurement principle involves laser and photodiode noise for OBD readout and resistor noise for piezoresistive strain-sensing readout. Laser noise in OBD readout (labelled as number 2 in Fig. 1a) comes from both fluctuations in the laser beam intensity and the spatial distribution, and from laser mode hopping^4^,^47^. Photodiode shot noise (labelled as number 3 in Fig. 1a) comes from statistical fluctuations in the number of photons emitted by the laser. For a well-designed system, this noise is usually dominant in OBD readout and it sets the lower limit for the deflection noise^4^,^47^. The inherent noise for piezoresistive strain-sensing readout is the resistor noise in the Wheatstone bridge. It includes both 1/*f* and Johnson resistor noise (labelled as number 3 in Fig. 1b). For the frequencies of interest in AM-AFM, Johnson noise usually predominates.

Finally, in both readouts there are also noise sources coming from the measurement electronics. In OBD readout electronics, the main noise sources are voltage and current noise of the transimpedance amplifier (labelled as numbers 4 and 5 in Fig. 1a), the transimpedance feedback resistor noise (labelled as number 6 in Fig. 1a) and the voltage divider noise (where the voltage divider is used in subsequent signal processing). In piezoresistive strain-sensing readout electronics, the main noise sources are noise of the bridge voltage reference (labelled as number 3 in Fig. 1b) and the voltage and current noise of the differential amplifier (labelled as number 4 and 5 in Fig. 1b).

## AFM imaging noise measurement procedure and obtained data

**Figure 4:**
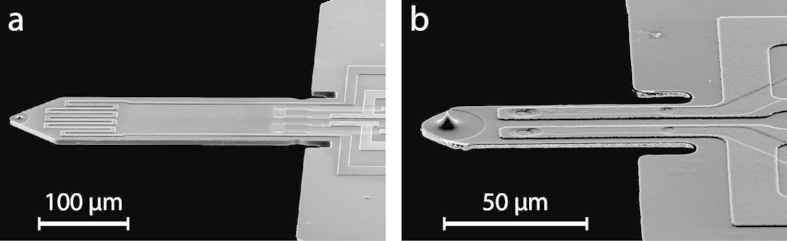
SEM images of representative (**a**) 300 × 100 μm and (**b**) 70 × 30 μm silicon piezoresistive cantilevers used for noise measurements and AFM imaging. (**a**) The large-sized piezoresistive cantilever has a meander-like patterned heater resistor for thermal actuation (close to the free end, not used in measurements) and two active piezoresistors (close to the fixed end). (**b**) The small-sized piezoresistive cantilever has two active piezoresistors along its length.

We measured our AFM system noise for the same cantilever using both OBD and piezoresistive readout. The cantilevers used in the measurements were 300 × 100 μm and 70 × 30 μm sized piezoresistive silicon cantilevers (PRSA and PRS probes, SCL-Sensor.Tech. Fabrication GmbH, Austria) presented in Fig. 4a,b. These cantilevers have a thickness from 4–6 μm and a resonance frequency around 80 kHz (300 × 100 μm) and 850 kHz (70 × 30 μm). The measured mechanical bandwidth of these cantilevers is around 0.8 kHz (300 × 100 μm) and 3 kHz (70 × 30 μm). We performed all measurements using a Bruker MultiMode 8 AFM system. We custom-made a cantilever holder enabling simultaneous optical and electrical readout to adapt into the MultiMode AFM head (see Supplementary Section II). A stack piezo actuator (PL022.30, Physik Instrumente, USA) integrated in the holder excited the cantilever resonance. The backside of the silicon cantilevers was sufficiently reflective for OBD measurements such that no reflective coating was required.

**Figure 5:**
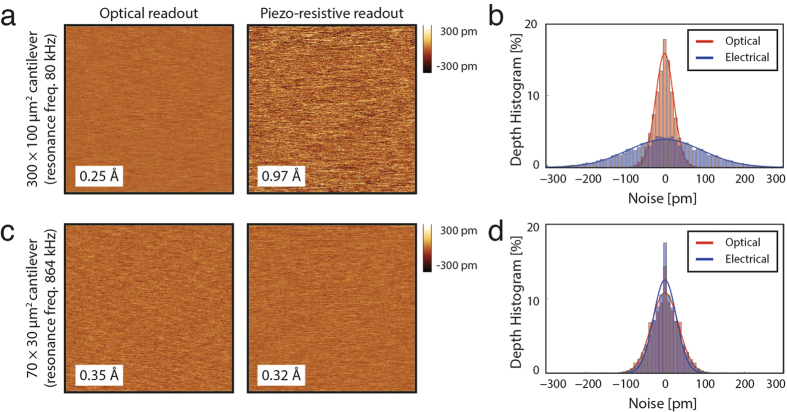
Noise measurements with OBD and piezoresistive readout. 300 × 100 μm piezoresistive cantilever: (**a**) Deflection noise measured with OBD and piezoresistive readout and (**b**) the corresponding noise histograms. 70 × 30 μm piezoresistive cantilever: (**c**) Deflection noise measured with the OBD and piezoresistive readout and (**d**) the corresponding noise histograms.

We characterised the RMS AFM imaging noise using 2D “noise images” in AM-AFM mode with each cantilever, using both the OBD and piezoresistive readout (see methods section for details). Figures 5a,c present the noise images, and Fig. 5b,d present the corresponding noise histograms.

For the large-sized cantilevers (300 × 100 μm), we used a lock-in measurement bandwidth of 4.8 kHz, a free amplitude of 50 nm and an amplification gain for the piezoresistive readout of 1000. Such large amplitude was necessary as these cantilevers exhibit a very strong long-range damping and high surface adhesion. For the small-sized cantilevers (70 × 30 μm) we used a lock-in measurement bandwidth of 20 kHz, a free amplitude of 20 nm and an amplification gain for the piezoresistive readout of 100. As expected, the imaging noise of the large-sized cantilevers with the piezoresistive readout was several times higher (around 1 Å) than it was with the OBD readout (0.25 Å). On the other hand, the imaging noise of the small-sized cantilevers measured with the piezoresistive readout was 0.32 Å, while the noise obtained with the OBD readout was 0.35 Å. Therefore, at the measurement bandwidth of 20 kHz we achieved with both readouts a deflection noise ≈0.3 Å, which is the Z noise level specified for the Bruker Multimode 8 AFM using AM-AFM mode in air at zero scan size^48^. To verify these results, we performed noise measurements with several small-sized cantilevers.

### AFM imaging with piezoresistive readout

**Figure 6:**
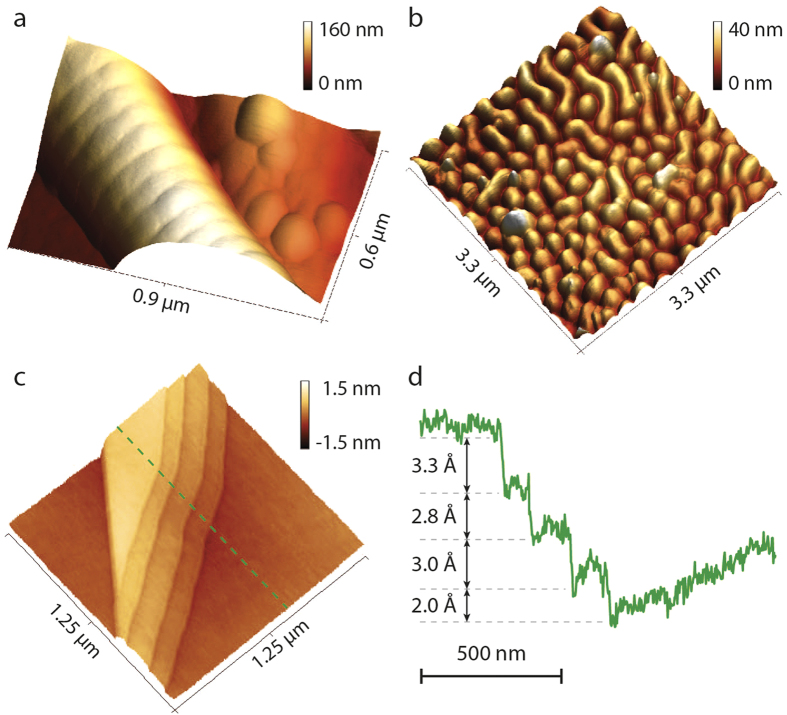
AFM images of biological and solid state samples obtained using piezoresistive readout. (**a**) An AFM image of a collagen fibril showing the characteristic 67 nm spaced bending pattern. (**b**) An AFM image of a housefly eye corneal surface pattern showing ~10 nm high features (**c**) An AFM image of a graphite (HOPG) surface, showing atomic steps. (**d**) The selected topography line demonstrates discernible topography features of order of 2 Å.

In order to show image quality achievable with piezoresistive readout, we used small-sized piezoresistive cantilevers to image several AFM samples with very low topography features (see Fig. 6a–c). We obtained all AFM images using AM-AFM imaging mode in air at 1 Hz scan rate. Figure 6a presents an AFM image of a collagen fibril showing the characteristic 67 nm spaced bending pattern. The collagen fibril corrugation depth is only a few nanometres. Figure 6b presents an AFM image of a housefly eye corneal surface pattern, showing maze-like features of only ~10 nm height. Figure 6c presents an AFM image of a highly ordered pyrolytic graphite (HOPG) showing graphite atomic steps. We chose a topography line (see Fig. 6c, the dashed green line), whose profile is presented in Fig. 6d. The line profile clearly shows that topography features of order 2 Å can be easily discerned using piezoresistive readout.

### Effect of the cantilever dimensions on different noise terms and achievable MDD

We performed theoretical calculations of the MDD achievable with both readouts, depending on the cantilever dimensions. The total deflection noise or MDD in both readouts can be expressed as


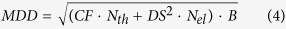


where *N*_*th*_ is the on-resonance thermomechanical noise power spectral density (PSD), *CF* is a correction factor determined for each readout (see Supplementary Sections IV and VI for details); *DS* is the deflection sensitivity of the readout (in units of distance per Volts or distance per Amperes); *N*_*el*_ represents the entire electrical noise PSD (coming from both measurement principle noise terms and the readout electronics), and *B* is the lock-in measurement bandwidth, usually set close to the mechanical bandwidth of the cantilever 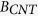. Although in AM-AFM mode the amplitude sensitivity, rather than deflection sensitivity should be used for scaling of the electrical noise 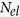, we assume that for stiff samples in air the deflection and amplitude sensitivity have very close values^49^,^50^,^51^. This assumption applies to the exact imaging conditions we propose for piezoresistive readout.

**Figure 7:**
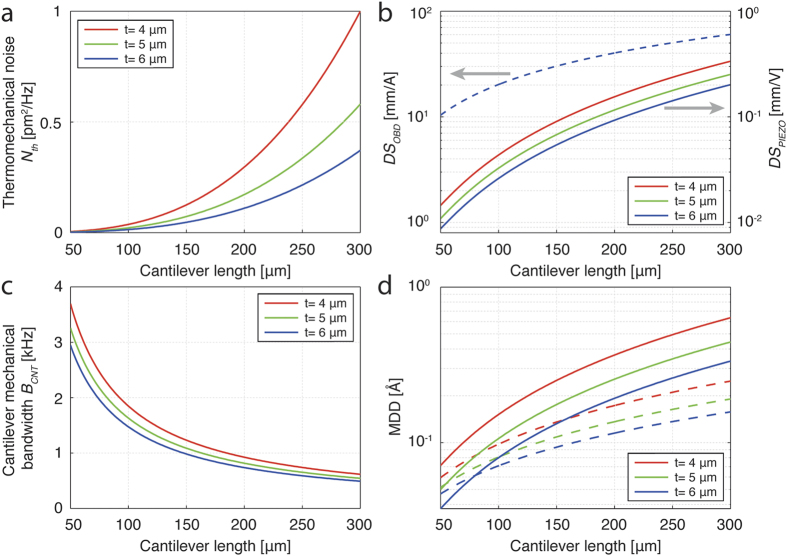
The effect of cantilever dimensions on terms contributing to deflection noise and achievable MDD, for both OBD and piezoresistive strain-sensing readout. (**a**) On-resonance thermomechanical noise power spectral density of the cantilever free end irrespective of detection method. (**b**) Deflection sensitivity of the OBD readout 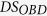 (dashed line, left axis in *mm/A*) and the piezoresistive readout 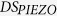 (full lines, right axis in *mm/V*). 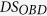 is independent of the cantilever thickness. (**c**) The cantilever mechanical bandwidth, estimated as 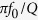. (**d**) A comparison of the theoretically achievable MDD, with OBD readout (dashed line) and piezoresistive readout (full lines), for several different cantilever thicknesses over the defined length range. The length-to-width ratio used is 70/30.

All terms in equation (4) depend on the cantilever geometry, except for 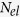, which remains constant. We estimated the influence of cantilever dimensions on the MDD and on the individual terms in equation (4), for both readouts (see Fig. 7a–d).

We compared the piezoresistive readout noise for small-sized cantilevers to the noise performance of a custom AFM head designed for OBD AFM imaging with small-sized cantilevers^52^. (See Supplementary Section III for OBD readout parameters.) We assumed that photodiode shot noise limits the total electrical noise of the OBD readout. We performed the noise calculations for the OBD readout using a procedure similar to that of Fukuma *et al.*^47^ (see Supplementary Section IV for details). For the case of piezoresistive readout we used the parameters estimated for 70 × 30 μm sized piezoresistive silicon cantilevers (PRS probes, SCL-Sensor.Tech. Fabrication GmbH, Austria). These cantilevers have two active and two passive p-type Boron doped piezoresistors which form a Wheatstone bridge (see Supplementary Section V for details). The noise calculations using the piezoresistive readout were performed using a procedure similar to one derived in^41^,^42^,^43^ (see Supplementary Section VI for details). In the calculations, we explored various cantilever geometries while keeping the piezoresistor dimensions and the doping properties constant. We varied the cantilever length from 50 to 300 μm with a constant length-to-width ratio at *l*/*w* = 70/30. We performed the calculations for three chosen cantilever thicknesses: 4 μm, 5 μm and 6 μm.

Figure 7a presents the on-resonance thermomechanical noise power spectral density of the cantilever free end 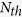, for a range of different cantilever dimensions. 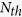 decreases for smaller lengths and larger thicknesses, corresponding to the cantilever geometries preferred for strain-sensing. Figure 7b presents the deflection sensitivity of the OBD readout and the piezoresistive readout. A dashed line indicates the OBD deflection sensitivity 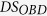 which does not depend on the cantilever thickness. Three solid lines show the piezoresistive deflection sensitivity 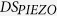, for three different cantilever thicknesses. Both deflection sensitivities are referred to the input of the first amplifying stage (the output of the photodiode for the OBD readout and the input of the Wheatstone bridge for the piezoresistive readout).

Lower values of the deflection sensitivity (distance units per Volts or Amperes) correspond to better performance of the readout. From Fig. 7b, we see that deflection sensitivity will improve with the cantilever length decrease, for both readouts. However, length decrease improves 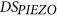 more significantly than 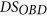. In addition, increasing the cantilever thickness further improves the performance of the piezoresistive readout.

The cantilever mechanical bandwidth 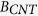 (estimated as 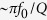, see Supplementary Section VII for details) increases with shrinking cantilever dimensions (see Fig. 7c). A cantilever with higher 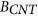 will respond more quickly to topography changes and allow for faster AM-AFM imaging. A higher 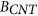 will also allow for a higher lock-in measurement bandwidth *B*. Although this permits faster AM-AFM detection, a higher *B* also increases the deflection noise. However, if faster scanning is not required, choosing a lower measurement bandwidth will decrease the imaging noise.

Even for a lock-in measurement bandwidth *B* set close to 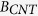, the overall achievable MDD still decreases with a decrease of the cantilever length (see Fig. 7d). The results presented in Fig. 7d suggest a set of cantilever dimensions, at which point the piezoresistive strain-sensing readout MDD equals the MDD of the OBD readout. After this point, piezoresistive readout performs better than the OBD readout in achievable MDD. Although 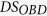 is independent of cantilever thickness, both the cantilever thermomechanical noise 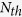 and the cantilever mechanical bandwidth 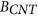 depend on thickness; therefore, a MDD achievable with OBD readout also depends on the cantilever thickness.

Any decrease in cantilever length and increase in thickness (at constant *l*/*w* ratio) inevitably leads to an increase of the spring constant. For cantilever dimensions we analysed, the spring constants range from 10s N/m to 100s N/m (see Supplementary Section VIII for details). The required cantilever spring constant for AFM depends strongly on the application and the imaging mode and can span up to four orders of magnitude. For AM-AFM imaging in air, cantilevers usually have spring constants in the range from few N/m up to 100 N/m. Traditionally, cantilevers with spring constants in hundreds N/m are considered very stiff and unsuitable for imaging soft samples, and are often identified with a rapid tip wear. However, tip wear comes mainly from lateral forces occurring when the feedback loop cannot compensate surface topography fast enough. A stiffer cantilever with a higher resonance frequency and a higher mechanical bandwidth enables a faster feedback, and hence mitigates the negative effect of the high spring constant on the tip wear. Furthermore, in certain AFM applications, *k* of the order of several hundred to several thousand is desired and successfully used^53^. Stiffer cantilevers also avoid the problem of surface adhesion that limits the use of softer cantilevers for AM-AFM imaging in air. Therefore, despite higher spring constants, cantilevers with the proposed dimensions are well suited for AM-AFM imaging of stiff samples in air.

## Discussion

Because piezoresistive sensors measure the strain in the cantilever they are best suited for cantilevers with larger thickness and higher spring constants. While this type of cantilever is not well suited for force sensing, we demonstrated that it is very well suited for deflection sensing and imaging of small feature topographies.

In this paper, we discussed the cases of two specific electronic readouts: an OBD readout using a transimpedance amplifier and voltage arithmetic electronics, and a piezoresistive readout using a Wheatstone bridge and a differential amplifier. Other types of electronic signal processing also exist for both OBD^54^ and piezoresistive^55^ readout. However, the electronic readouts that we have chosen to analyse are up to present the most commonly used ones.

The low noise instrumentation amplifier we used in our measurements and calculations (see the methods section) has a 4 MHz bandwidth at 10×  gain. A wide range of cantilevers with geometries where piezoresistive readout outperforms OBD readout have resonance frequencies within this bandwidth. For further improvement (e.g. higher frequencies), alternate readout setups should also be investigated^55^.

An additional two-fold increase in the piezoresistive readout signal could be achieved by incorporating four active piezoresistors on the cantilever, with two piezoresistors each on both the tensile and compressive sides respectively. However, the fabrication of such a cantilever would become very challenging.

We showed that with a measurement bandwidth of 20 kHz and an estimated resistor power dissipation of around 2 mW, the measured imaging noise with the piezoresistive readout is only ≈0.3 Å. However, it is the tip temperature, rather than overall cantilever dissipation that is critical in AFM. We simulated the cantilever heating and concluded that the tip temperature was on par with the temperatures occurring in OBD readout.

We didn’t discuss the application of the proposed piezoresistive cantilevers for measurements in fluids. Even though imaging in fluid with piezoresistive cantilevers is possible^56^,^57^, stiff cantilevers are generally not well suited for imaging of soft biological samples, which are the most common samples that demand a fluid environment.

Although miniaturisation improves cantilever performance, shrinking cantilever dimensions becomes problematic for both readout methods. For OBD readout, cantilever dimensions close to the optical diffraction limit result in laser light spill over and a loss of signal. On the other hand, in piezoresistive readout, fabrication of shallow piezoresistors (necessary to maintain signal-to-noise performance) is very challenging^20^,^21^. While the former issue is fundamental, the latter issue is addressable through further developments in the fabrication process of piezoresistive cantilevers.

### Outlook

In this paper, we demonstrated, both theoretically and experimentally that small-sized cantilevers can have equal or better AFM imaging noise performance using piezoresistive readout than using OBD readout. This result refutes the common belief that self-sensing cantilevers are always noisier than optically detected cantilevers. For AM-AFM imaging in air, small-sized and high spring constant cantilevers offer a viable alternative to optical beam deflection. They enable a whole set of different applications where OBD readout is either not practical or not possible. In the future, further improvements in microfabrication and development of improved strain sensing materials may allow miniaturisation of AFM cantilevers below the optical diffraction limit. Such ultra-miniature cantilevers will further increase the sensitivity and speed of cantilevers for next generation high-speed AFMs. Therefore, we hope that this result stimulates further advances in miniaturisation of piezoresistive self-sensing cantilevers.

## Methods

### Piezoresistive readout

The electrical piezoresistive readout setup was custom designed and fabricated. It consisted of an instrumentation amplifier and additional amplification stages. Two active piezoresistors integrated on the cantilever body and two passive piezoresistors integrated on the cantilever chip formed a Wheatstone bridge used for piezoresistive readout. The bridge resistance was around 1 kΩ, for both large (300 × 100 μm) and small (70 × 30 μm) sized cantilevers. A 2 V input voltage was supplied to the bridge. The output signals from the bridge were sent to a low noise instrumentation amplifier AD8429 (Analog Devices, USA) and afterwards signal was amplified by additional amplification stages. The total amplification gain of the measured electrical signal is switchable to either 100 or 1000. The amplified signal was input to the Bruker AFM Nanoscope controller as the deflection signal at the *IN0* input of the signal access module.

### Noise measurement procedure

We performed all noise measurements in AM-AFM. In order to obtain the 2D “noise image”, we performed the following procedure: first, we set the AFM image scan size to a value small enough that the tip can be considered as not moving and that there is no change in the surface topography (e.g. 0.01 nm). Then, the proportional and integral gains of the AFM PI controller were set to a very small value, just to prevent the cantilever from drifting off the surface. As the gains are set so low, almost the entire signal obtained from the “surface topography” is present in the amplitude error image. Since we consider that there is no change in surface topography, we can assume that the entire amplitude error signal actually represents noise present in the system. The amplitude error images taken in volts are scaled by the measured amplitude sensitivity parameter in order to obtain a 2D image of the noise in distance units. Using AFM image processing software^58^ and processing the distribution of the pixel heights in the 2D noise image an RMS value of the noise was derived.

### AFM imaging and sample preparation

We performed all AFM imaging in AM-AFM mode using a custom made piezoresistive cantilever holder with readout electronics in combination with a commercial AFM system (Bruker MultiMode 8). The images were taken in air at 1 Hz scan rate and at resonance frequencies in the range of 840–860 kHz. We extracted collagen from a rat tail tendon as described in^59^. To prepare the corneal sample from a captured housefly, we dissected the head from the body with a scalpel, and afterwards an eye from the head in the same fashion. We used a scalpel to break the eye into several pieces, and some pieces were attached to an AFM sample disc via double-sided sticky tape. A freshly cleaved HOPG sample was prepared by cleaving a block of HOPG (PFQNM-SMPKIT-12M, Bruker, USA) with a sticky tape.

### AFM image processing

AFM images were processed using Gwyddion^58^. We used standard AFM image processing steps: levelling sample tilt by plane subtraction, removing scanner bow by 2D polynomial fitting, line-by-line matching of height median and line-by-line linear fitting. Lastly, some images are presented as pseudo-three-dimensional images.

## Additional Information

**How to cite this article**: Dukic, M. *et al.* Piezoresistive AFM cantilevers surpassing standard optical beam deflection in low noise topography imaging. *Sci. Rep.* **5**, 16393; doi: 10.1038/srep16393 (2015).

## Supplementary Material

Supplementary Information
