# Supplementary material for: Piezoresistive AFM cantilevers surpassing standard optical beam deflection in low noise topography imaging
Source: Sci Rep. 2015 Nov 17;5:16393. doi: 10.1038/srep16393 (PMC4647226; doi:10.1038/srep16393)
Supplement: Supplementary Information [file srep16393-s1.pdf]

SUPPLEMENTARY INFORMATION

Piezoresistive AFM cantilevers surpassing standard optical beam detection in low noise topography imaging

Maja Dukic, Jonathan D. Adams and Georg E. Fantner

Contents

I Dependence of cantilever bending angle on a cantilever free end deflection 1
II Measurement setup 2
III Estimated parameters for the OBD readout 2
IV MDD derivation for OBD readout 3
V Estimated parameters for the piezoresistive readout 5
VI MDD derivation for piezoresistive readout 6
VII Estimation of the cantilever mechanical bandwidth 7
VIII Dependence of the cantilever spring constant on the cantilever dimensions 7
IX References 8

I Dependence of cantilever bending angle on a cantilever free end deflection

A cantilever deflection Δz and bending angle θ along the cantilever length, coming from a point load F acting on the free end, are[1]

$$\Delta z(x) = \frac{Fl^3}{6EI} \cdot \left[3\left(\frac{x}{l}\right)^2 - \left(\frac{x}{l}\right)^3\right] \tag{S1}$$

$$\theta(x) = \frac{Fl^2}{2EI} \cdot \left[2\left(\frac{x}{l}\right) - \left(\frac{x}{l}\right)^2\right] \tag{S2}$$

where l is the cantilever length, x is the position along the cantilever length (starting from the fixed end), E is Young's modulus of the cantilever material along its length and I is the moment of inertia of the cantilever cross section about its neutral axis. The laser beam used in OBD readout is a Gaussian beam, and it is common to define the laser beam diameter as a point where the laser intensity falls to a fraction $1/e^2$ of its initial intensity. We will denote l_b as the laser beam diameter along the cantilever length. In order to reflect most of the laser power off of the cantilever surface, the optimal position of the center of the laser beam spot, along the cantilever length is $x_o \approx l - l_b/2$. Inputting x_o in equation (S2) and expressing it in terms of $\Delta z(l)$ we get the equation

$$\theta\left(l - \frac{l_b}{2}\right) = \frac{3}{2l}\left(1 - \left(\frac{l_b}{2l}\right)^2\right) \cdot \Delta z(l) \tag{S3}$$

II Measurement setup

AFM imaging and noise measurements were performed using a custom made cantilever holder (see Figure S1a-c). The cantilever holder was designed in order to enable simultaneous measurements with both the OBD and the piezoresistive readout. A stack piezo actuator (PL022.30, Physik Instrumente, USA) was integrated in the holder to excite the cantilever resonance. The custom made electronics setup was used for electrical readout of the self-sensing cantilevers. The electrical readout consisted of a full Wheatstone bridge of piezoresistors located on the cantilever chip and subsequent amplification stages (see Figure S1d). The flexible printed circuit board (PCB) was used to provide signals to and from the cantilever chip. A low noise instrumentation amplifier AD8429 (Analog Devices, USA) was positioned on the flexible PCB, close to the cantilever chip to reduce noise and stray capacitances of the electrical lines. The rest of the amplification stages were located on the readout electronics PCB. An ultra-precision, low noise voltage reference ADR420 (Analog Devices, USA) was used to bias the Wheatstone bridge.

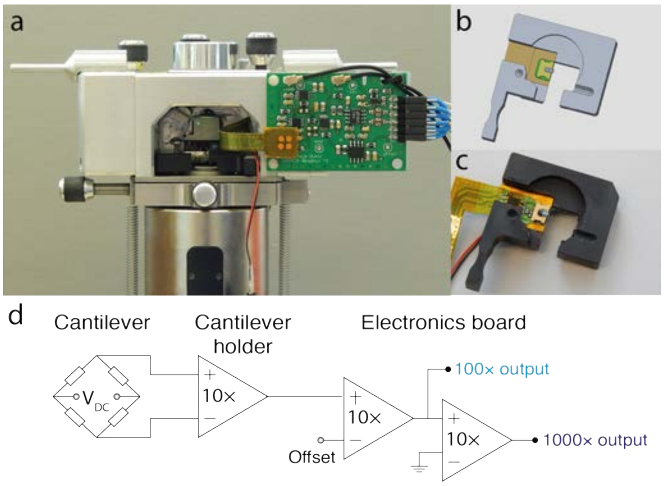

Figure S1. *a) AFM head with custom designed cantilever holder and readout electronics b) The design and c) an image of the cantilever holder designed for simultaneous measurements with both OBD and electrical readout. d) Schematic of the electrical readout.*

III Estimated parameters for the OBD readout

For the case of the OBD readout, parameters for a custom made optical AFM head designed for AFM imaging with the small sized cantilevers[2] are given in Table S1. All calculations were performed for room temperature $T = 23\ C^o$.

Table S1. *Estimated OBD readout parameters*

Parameter	Value
Laser spot width: w_b $[\mu m]$	5.8
Laser spot length: l_b $[\mu m]$	18
Lens focal length: l_f $[mm]$	4.6
Diameter of the collimated laser beam: a_0 $[mm]$	2.5
Laser power: P_0 $[mW]$	3
Photodiode responsivity: η $[A/W]$	0.42
Laser light optical path loss attenuation factor: α_1	0.8
Laser light spillage attenuation factor: α_2	$erf(2\sqrt{2}w/w_b)$
Laser light Si absorption attenuation factor: α_3	0.35
Total laser light attenuation factor: α	$\alpha_1 \cdot \alpha_2 \cdot \alpha_3$
Transimpedance feedback resistance: R_F $[k\Omega]$	20

IV MDD derivation for OBD readout

The overall deflection noise of OBD readout can be calculated by adding deflection power spectral densities (PSDs) of all relevant noise sources and then integrating this sum over the frequency range of an AFM lock-in measurement bandwidth. To perform this calculation, the deflection sensitivity of the OBD readout method also needs to be determined in order to scale electrical noises from amperes to distance units.

The mean square deflection at the cantilever free end, for fundamental resonance mode, as seen with OBD readout is[3–5]

$$\widehat{z_1^2} = \frac{16}{3\alpha_1^2} \cdot \left(\frac{\sin\alpha_1 \sinh\alpha_1}{\sin\alpha_1 + \sinh\alpha_1}\right)^2 \cdot \frac{k_B T}{k} = 0.8175 \cdot \frac{k_B T}{k} \tag{S4}$$

where k_B is the Boltzmann constant, T is the temperature and $\alpha_1 = 1.875$ for the first resonance mode. Equation (S4) differs from equation (3) given in the paper because OBD readout measures angular changes, rather than deflection, so the correction factor is introduced[3–5]. From equation (S4) and the cantilever amplitude transfer function[6] we obtain the deflection noise PSD of the first resonance mode of the cantilever:

$$S_z(f) = 0.8175 \cdot \frac{4k_B T}{2\pi f_0 k Q} \cdot \frac{1}{\left(1 - \left(\frac{f}{f_0 Q}\right)^2\right)^2 + \left(\frac{f}{f_0 Q}\right)^2} \tag{S5}$$

where f_0 and Q are the cantilever fundamental mode resonance frequency and the quality factor, respectively. Assuming that we excite the cantilever oscillations at the frequency $f_e \cong f_0$ and that the lock-in measurement bandwidth is B, we obtain the power of the deflection noise coming from the thermomechanical noise[7,8]:

$$N_{th,B} \cong 0.8175 \cdot \frac{2k_B T Q B}{\pi f_0 k} \tag{S6}$$

In the OBD readout electronics, a transimpedance amplifier is commonly used as the first stage amplifier, to convert the photodiode current I_D to voltage. Noise sources present in this readout include the photodiode current shot noise $i_{N,D}$, the amplifier input referred current noise $i_{N,amp}$ and the feedback resistor noise $i_{N,R}$. These noise sources can all be treated identically as the current noise sources, which add onto the measured photodiode current[9]. These noise sources have the same gain, which is constant in the amplifier flat-band.

Additional noise source also present in the readout is the influence of the amplifier noninverting input voltage noise $e_{N,amp}$. This noise, contrary to other noise sources, varies with frequency in the amplifier flat-band. $e_{N,amp}$ is multiplied by the amplifier's non-inverting closed loop gain $A_{CL}(f)$. The referred-to-input (RTI) voltage amplifier noise $e_{N,amp}$ sees the amplifier circuit as presented in Figure S2, where $Z_F = R_F || C_F$ represents the total feedback impedance and C_S represents the total stray capacitance which includes the photodiode capacitance, the amplifier input capacitance and other stray capacitances (e.g. such as the ones coming from the traces).

If $A_{OL}(j\omega)$ is the amplifier open loop gain frequency response, then $e_{N,\square mp}$ is amplified by the amplifier's non-inverting closed loop gain:

$$A_{CL}(j\omega) = \frac{A_{OL}(j\omega)}{1 + \frac{A_{OL}(j\omega)}{1 + j\omega C_S Z_F}} \tag{S7}$$

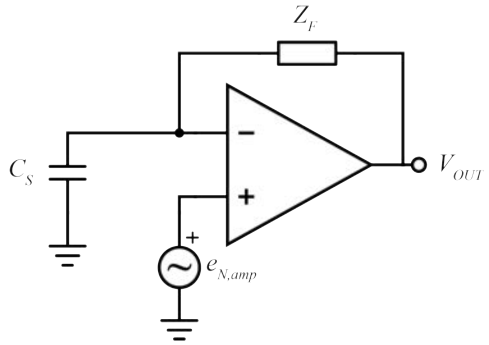

Figure S2. *The amplifier circuit seen by the RTI amplifier voltage noise* $e_{N,amp}$

In the amplifier flat-band we can assume that

$$A_{CL}(j\omega) \cong 1 + j\omega C_S Z_F \tag{S8}$$

By substituting $Z_F = R_F/(1 + j\omega R_F C_F)$ in equation (S7) and assuming that $C_S \gg C_F$ we obtain

$$A_{CL}(j\omega) \cong \frac{1 + j\omega R_F C_S}{1 + j\omega R_F C_F} \tag{S9}$$

From (S9) we see that the closed loop gain transfer function has a zero at $f_z = 1/2\pi R_F C_S$ (where the gain starts to rise) and a pole at $f_p = 1/2\pi R_F C_F$ (where the gain levels off). For low frequencies $A_{CL}(f) \approx 1$ which will result in the input referred current noise of $e_{N,amp}/R_F$.

Finally, the total input referred current noise PSD of the transimpedance readout in the gain flat-band can be written as

$$N_i = 2eI_D + i_{N,amp}^2 + \frac{4k_B T}{R_F} + \left(|A_{CL}(j\omega)| \cdot \frac{e_{N,amp}}{R_F}\right)^2 \tag{S10}$$

where e is an electron charge and R_F is the resistance of the feedback resistor. In equation (S10) we take into account the entire current of the photo sensitive detector I_D but for the rest of the noise sources we consider only noise sources coming from one transimpedance amplifier. Depending on the number of photodiode quadrants n_{PD} in the detector, the remaining noise terms need to be multiplied by n_{PD} to obtain the total readout noise. Finally, the current noise needs to be scaled to distance units by the deflection sensitivity (in nm/A units). The deflection sensitivity of the OBD readout is[10]

$$DS_{OBD} = \frac{\Delta z}{\Delta I} = \frac{l a_0}{6\eta\chi\alpha P_0 l_f} \cdot \frac{1}{1 - \left(\frac{l_b}{2l}\right)^2} \tag{S11}$$

where a_0 is the diameter of the short axis of the collimated laser beam, l_f is the focal length of the focusing lens, η is the efficiency of the light-to-current conversion at the photodiode, χ is the correction factor correcting for the assumed rectangular shape of the laser spot[10], P_0 is the laser power and α is the total laser power attenuation factor (coming from the optical path loss, the laser light spillage and the cantilever absorption). Finally, the total deflection noise or MDD of OBD readout equals to

$$n_{OBD} = \sqrt{N_{th,B} + DS_{OBD}^2 \cdot B \cdot N_i} \tag{S12}$$

In this section, we ignored laser noise sources for two main reasons: the laser intensity fluctuations are mostly eliminated by the differential amplifier present in the OBD readout electronics, and laser mode fluctuations that cause fluctuations in spatial distribution are very hard to estimate and strongly depend on the OBD setup[11].

Finally, in most cases the noise term coming from the photodiode shot noise will be the dominant one ($N_i \approx 2eI_D$) and for any well designed system it will determine the lower limit of deflection noise[10,11]. Therefore, we used this assumption in the noise calculations presented in the paper.

V Estimated parameters for the piezoresistive readout

For the case of the piezoresistive readout, the estimated readout parameters for the 70×30 μm sized piezoresistive silicon cantilevers (PRS probes, SCL-Sensor.Tech. Fabrication GmbH, Austria) are given in Table S2. The differential amplifier used in the calculations was the low noise instrumentation amplifier AD8429 (Analog Devices, USA), set to a gain of 10. This amplifier was also used in the noise measurements and AFM imaging. All calculations were performed for room temperature $T = 23\ C^o$.

Table S2. *Estimated piezoresistive readout parameters*

**Parameter**	**Value**
Active piezoresistor length: l_s $[\mu m]$	40
Piezoresistor width: w_s $[\mu m]$	5
Piezoresistor thickness: t_s $[\mu m]$	1
Doping concentration: c $[cm^{-3}]$[12]	8.5×10^{18}
Piezoresistor resistance: ρ_R $[\Omega\ cm]$	0.01
Longitudinal piezoresistive coefficient: π_l $[Pa^{-1}]$[13]	4.72×10^{-10}

VI MDD derivation for piezoresistive readout

The deflection noise of piezoresistive strain-sensing readout can be calculated in similar fashion as was the case for the OBD readout. To perform this calculation, the deflection sensitivity of the piezoresistive readout method needs to be determined in order to scale the electrical noises from volts to distance units.

The mean square deflection at the free end of the cantilever for the fundamental resonance mode, as seen with piezoresistive readout is[14]

$$\widehat{z_1^2} = \frac{4}{3} \cdot \frac{k_B T}{k} \tag{S13}$$

Equation (S12) differs from equation (3) given in the paper because piezoresistive strain-sensing readout measures changes in the induced strain, rather than deflection, so a correction factor is introduced. Assuming that we excite cantilever oscillations at a frequency $f_e \cong f_0$ and that the lock-in measurement bandwidth is B, we obtain the power of the deflection noise coming from the thermomechanical noise:

$$N_{th,B} = \frac{4}{3} \cdot \frac{2k_B TQB}{\pi f_0 k} \tag{S14}$$

The PSD of the piezoresistor Johnson noise, from the Wheatstone bridge is $N_w = 4k_B TR$. Johnson noise coming from the differential amplifier is

$$N_{amp} = e_{N,amp}^2 + \frac{i_{N,amp}^2 R^2}{2} \tag{S15}$$

where $e_{N,amp}$ and $i_{N,amp}$ are the input-referred Johnson voltage and current noise of the amplifier. Finally, some of the noise coming from the bridge voltage reference will affect the readout, where the level of the influence depends on the common mode rejection ratio (CMRR) of the differential amplifier. The PSD of the bridge voltage reference noise, referred to the amplifier input is

$$N_{ref} = \frac{n_{ref}^2}{4 \cdot 10^{CMRR_{dB}/10}} \tag{S16}$$

where $CMRR_{dB}$ is the amplifier CMRR expressed in decibels and n_{ref} is the voltage noise spectral density of the bridge voltage reference. Usually, with a well-chosen differential amplifier and a low noise bridge reference (e.g. such as battery), this noise term is negligible. The total electrical noise PSD is then

$$N_v = N_w + N_{amp} + N_{ref} \tag{S17}$$

In order to calculate the total deflection noise, the electrical noise needs to be scaled by the deflection sensitivity (in nm/V units, calculated for the case of two active resistors on the cantilever)[14–16]

$$DS_{PIEZO} = \frac{\Delta z}{\Delta V} = \frac{4}{3} \cdot \frac{l^2}{E(t - t_s)\left(1 - \frac{l_s}{2l}\right)} \cdot \frac{1}{\pi_l V_B} \tag{S18}$$

where l and t are the cantilever length and thickness, l_s and t_s are the piezoresistor length and thickness, E is Young's modulus of the cantilever material, along its length, π_l is the longitudinal piezoresistive coefficient, and V_B is the bridge supply voltage. Finally, total deflection noise or MDD of the piezoresistive readout equals to

$$n_{OBD} = \sqrt{N_{th,B} + DS_{PIEZO}^2 \cdot B \cdot N_v} \tag{S19}$$

VII Estimation of the cantilever mechanical bandwidth

We estimated the cantilever mechanical bandwidth as $\pi f_0/Q$ where f_0 and Q are the first mode resonance frequency and the quality factor, respectively. f_0 was calculated as[17]

$$f_0 = \frac{1.875^2}{2\pi} \cdot \frac{t}{l^2} \cdot \sqrt{\frac{E}{12\rho}} \tag{S20}$$

where l and t are the cantilever length and thickness, E is the Young's modulus of the cantilever material along the cantilever length and ρ is the cantilever material density. The quality factor Q in air was calculated as[18,19]

$$Q = \frac{4\rho t w f_0}{6\eta + 3w\sqrt{\eta (M/RT)\pi f_0 p}} \tag{S21}$$

where w is the cantilever width, R is the gas constant, and η, M, T and p are air dynamic viscosity, molar mass, temperature and pressure, respectively.

VIII Dependence of the cantilever spring constant on the cantilever dimensions

The spring constants of the analysed cantilever dimensions are presented in Figure S3.

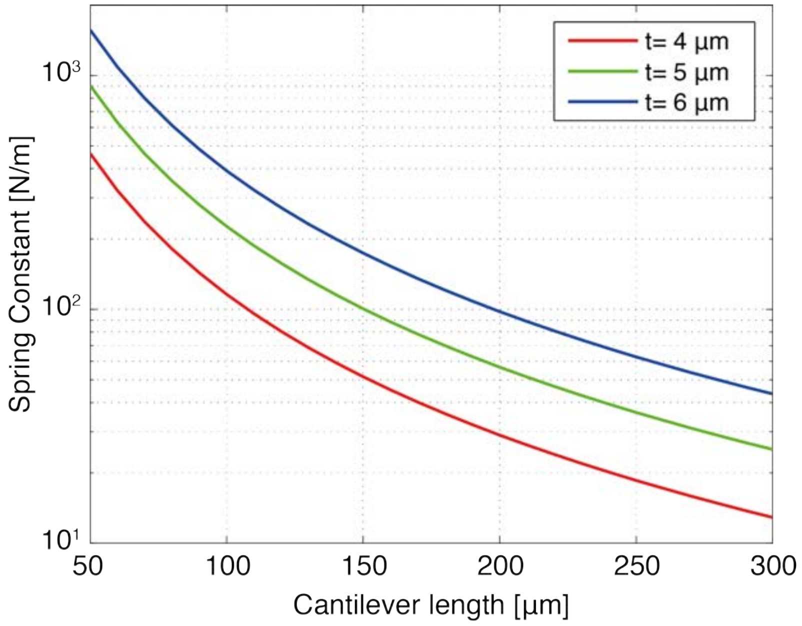

Figure S3. *Dependence of cantilever spring constant on the cantilever dimensions. The cantilever length-to-width ratio is kept constant at $l/w = 70/30$.*

IX References

1. Gere, J. M. & Goodno, B. J. *Mechanics of materials*. (Cengage Learning, 2009).
2. Adams, J. D. *et al.* High-speed imaging upgrade for a standard sample scanning atomic force microscope using small cantilevers. *Rev. Sci. Instrum.* **85,** 093702 (2014).
3. Butt, H.-J. & Jaschke, M. Calculation of thermal noise in atomic force microscopy. *Nanotechnology* **6,** 1–7 (1995).
4. Lévy, R. & Maaloum, M. Measuring the spring constant of atomic force microscope cantilevers: thermal fluctuations and other methods. *Nanotechnology* **13,** 33–37 (2002).
5. Ohler, B. Cantilever spring constant calibration using laser Doppler vibrometry. *Rev. Sci. Instrum.* **78,** 063701 (2007).
6. Shusteff, M., Burg, T. P. & Manalis, S. R. Measuring Boltzmann's constant with a low-cost atomic force microscope: An undergraduate experiment. *Am. J. Phys.* **74,** 873–879 (2006).
7. Garcia, R. *Amplitude modulation atomic force microscopy*. (WILEY-VCH, 2010).
8. Ando, T., Uchihashi, T. & Fukuma, T. High-speed atomic force microscopy for nano-visualization of dynamic biomolecular processes. *Prog. Surf. Sci.* **83,** 337–437 (2008).
9. Hobbs, P. C. D. Photodiode Front Ends: The Real Story. *Opt. Photonics News* **12,** 42–45 (2001).
10. Fukuma, T. & Jarvis, S. P. Development of liquid-environment frequency modulation atomic force microscope with low noise deflection sensor for cantilevers of various dimensions. *Rev. Sci. Instrum.* **77,** 043701 (2006).
11. Fukuma, T., Kimura, M., Kobayashi, K., Matsushige, K. & Yamada, H. Development of low noise cantilever deflection sensor for multienvironment frequency-modulation atomic force microscopy. *Rev. Sci. Instrum.* **76,** 053704 (2005).
12. ASTM Standard F 723, 1999, Standard Practice for Conversion Between Resistivity and Dopant Density for Boron-Doped, Phosphorous-Doped, and Arsenic-Doped Silicon. *ASTM international, West Conshohocken, PA (1999)*
13. Harley, J. A. & Kenny, T. W. 1/f noise considerations for the design and process optimization of piezoresistive cantilevers. *J. Microelectromechanical Syst.* **9,** 226–235 (2000).
14. Hansen, O. & Boisen, A. Noise in piezoresistive atomic force microscopy. *Nanotechnology* **10,** 51–60 (1999).
15. Park, S.-J., Doll, J. C. & Pruitt, B. L. Piezoresistive Cantilever Performance-Part I: Analytical Model for Sensitivity. *J. Microelectromech. Syst.* **19,** 137–148 (2010).
16. Doll, J. C. & Pruitt, B. L. Design of piezoresistive versus piezoelectric contact mode scanning probes. *J. Micromechanics Microengineering* **20,** 095023 (2010).
17. Sarid, D. *Scanning force microscopy*. (Oxford University Press, 1994).
18. Hosaka, H., Itao, K. & Kuroda, S. Damping characteristics of beam-shaped micro-oscillators. *Sensors Actuators A Phys.* **49,** 87–95 (1995).
19. Lübbe, J., Temmen, M., Schnieder, H. & Reichling, M. Measurement and modelling of non-contact atomic force microscope cantilever properties from ultra-high vacuum to normal pressure conditions. *Meas. Sci. Technol.* **22,** 055501 (2011).
